# Comparison of Physical, Occupational, and Sociocognitive Characteristics of Corporate and Private Taxi Drivers in Korea

**DOI:** 10.3390/healthcare9020224

**Published:** 2021-02-17

**Authors:** JongSun Ok, Hyeongsu Kim, Kyonghwa Kang

**Affiliations:** 1 Department of Nursing, Konkuk University, Chungju 27478, Korea; sokei@kku.ac.kr; 2 Department of Preventive Medicine, School of Medicine, Konkuk University, Seoul 05029, Korea; 3 Department of Nursing, Chungwoon University, Hongseong 32244, Korea; kh_kang@chungwoon.ac.kr

**Keywords:** occupational health, motor vehicle, health risk behavior

## Abstract

Taxis are a form of public transport which is very closely related to the safety of the public. Although private and corporate taxis have quite different characteristics, there have only been a few studies comparing the characteristics of corporate and private taxis. Moreover, among various characteristics, research was conducted mainly focusing on occupational characteristics. This study was undertaken to compare various physical, occupational, and sociocognitive characteristics of corporate and private taxi drivers. A descriptive cross-sectional study was conducted from 22 August to 11 September 2018. The subjects of this study were 960 corporate and private taxi drivers over 30 years old in Seoul to compare the means and association between private and corporate taxi drivers’ characteristics. In terms of the physical characteristics, corporate taxi drivers’ general physical health status was worse. In terms of the occupational characteristics, corporate taxi drivers had a high working intensity, and the incidence rate of traffic accidents and near misses was also high. This comparison of the characteristics of corporate and private taxis is expected to serve as evidence for developing tailored policies and programs to improve the health of corporate and private taxi drivers.

## 1. Introduction

Taxis are a form of public transport, and taxi drivers have unique physical, socioeconomic, occupational, and emotional characteristics that distinguish them from other employees. Compared to white-collar workers, taxi drivers complained of more musculoskeletal symptoms related to the shoulder and back, and the rate of psychological stress such as anxiety and agitation was higher [1]. In addition, taxi drivers reported having less sleep time than drivers of city buses, which represent a similar type of commercial vehicle, and they also reported that the driving time, driving distance, and number of near misses were higher [2]. In particular, taxi drivers are reported to have a greater burden in their efforts to achieve the target for daily taxi driving. This is because the wage of the taxi driver increases upon driving the taxi a longer distance within a specified time [2]. Taxi fares in Korea are only one-third of the fares based on distance and time compared to other Organization for Economic Cooperation and Development (OECD) countries. In addition, since taxi-only vehicles are not used, maintenance costs are high due to the purchase of commercial vehicles [3].

In 2020, the percentages of private taxis and corporate taxis in Korea were 65.8% and 34.2%, respectively. In other words, there are almost twice as many private taxis in Korea as corporate taxis [4]. This is the highest level compared to the percentages of private taxis by country as of 2012, which are 35% in Japan, 43% in the United Kingdom, and 42% in the United States [5]. Private taxis and corporate taxis have quite different characteristics. In order to qualify as a private taxi driver who can take over a private taxi, taxi drivers must have more than 5 years of accident-free driving, as well as corporate taxi driver experience. However, with the revision of the Enforcement Rules of the Passenger Transport Business Act in 2021, the standards for obtaining private taxi qualifications were eased, and it is now possible to obtain private taxi qualifications if you have 5 years of accident-free driving experience in a personal car [6]. Although the amount varies depending on the region, approximately KRW 80 million to 100 million is required for the purchase of a private taxi, and it is impossible to sell the acquisition within the next five years [6]. In the Korean health insurance system, private taxi drivers become self-employed health insurance members, and corporate taxi drivers become employee health insurance members because they belong to a taxi company. In the case of employee health insurance, insurance premiums are calculated only on the basis of monthly salary, while self-employed health insurance is calculated by considering monthly income and properties such as real estate and automobiles [7]. Therefore, private taxi drivers have a higher burden of health insurance premiums than corporate taxi drivers. Unlike private taxis, corporate taxis are required to make base payments to operate a taxi [8]. This refers to the taxi rental fee for 12 h a day. As the taxi fare increases, the base payments also increase. However, due to the taxi fare increase, taxi passengers reduce their use of taxis, and the income of taxi drivers decreases further. In addition, corporate taxi drivers have to work shifts in the morning and afternoon [8].

However, since there is more interest in traffic accidents and driving behaviors caused by taxis, most previous studies simply presented the characteristics of taxi drivers. In particular, only a few studies investigated the physical characteristics of taxi drivers regardless of the type of taxi driver (corporate or private) [8,9,10]. Furthermore, most of these studies only mentioned occupational characteristics among the various characteristics of taxi drivers, and they had a limitation in analyzing tachometer data [8,9,10]. In particular, there are limitations in the investigation of the social, economic, and emotional characteristics of taxi drivers with tachometer data analysis.

Unlike workers in other occupations who gather and work in certain places, taxi drivers spend a lot of time outside; thus, it tends to be difficult to gather information about their physical and psychosocial health-related status. However, since the physical and psychosocial health-related status of taxi drivers is very closely related to the safety of taxi passengers, it is important to investigate this to prepare measures to improve the health of taxi drivers. Therefore, in this study, we compared various physical, occupational, and sociocognitive characteristics of corporate and private taxi drivers in Seoul. This comparison of the characteristics of corporate and private taxis is expected to serve as evidence for developing policies and programs to improve the health of corporate and private taxi drivers.

## 2. Materials and Methods

### 2.1. Study Design and Subjects

This study is a descriptive cross-sectional study that compares physical, occupational, and sociocognitive characteristics between corporate taxi drivers and private taxi drivers. The subjects of this study were corporate and private taxi drivers over 30 years old driving taxis in Seoul.

### 2.2. Measurements and Variables

#### 2.2.1. Measurements

The survey questions were developed through meetings between the researchers and outside experts. According to existing research results on taxi drivers, this research questionnaire constituted a survey of taxi drivers’ personal history, basic data related to taxi driving, and questions on the health status of taxi drivers. Through this, we aimed to investigate various environmental risk factors for taxi drivers. To evaluate the adequacy of the developed questions, two groups of private taxi drivers and corporate taxi drivers were selected. A preliminary questionnaire was conducted with them, collecting their opinions, and the final questions were confirmed by revising and supplementing the questions.

#### 2.2.2. Variables

The questionnaire with a self-report form was composed of questions to understand the physical, occupational, and sociocognitive characteristics of corporate and private taxi drivers.

First, the physical characteristics variables were age, body mass index (BMI), physical health discomfort, duration of physical health discomfort over the last 2 weeks (days), general physical health status (very good, good, usual, bad, or very bad), the number and types of chronic disease, and the effects of chronic diseases on taxi driving (no effect, generally no effect, usually, mostly influences, or greatly influences). Chronic diseases were those diagnosed by physicians and currently being treated by regularly visiting a medical institution. As chronic diseases, diseases of the circulatory system, respiratory system, endocrine system, and musculoskeletal system were investigated.

The occupational characteristic variables were taxi driving work experience, number of working days per week, daily driving time, daily mileage, number of night shifts per month (working after 10 p.m.), number of traffic accidents or near misses in the last 3 years, and the relationship between traffic accidents (or near misses) and chronic diseases (not relevant, mostly not relevant, usually, mostly relevant, or very relevant). Near misses are cases in which a traffic accident may have occurred but did not lead to a direct traffic accident with personal and material damages.

The sociocognitive characteristics variables were marital status (unmarried, married with spouse, or married with no spouse (e.g., divorced)), number of dependent family members, monthly household income, income satisfaction (large enough, enough, average, insufficient, or very insufficient), and cognitive impairment. The Korean Dementia Screening Questionnaire (KDSQ) was used to evaluate cognitive impairment. This tool is a three-point scale composed of a total of 15 questions, with the score ranging from 0 to 30 points. The cutoff point of this tool is 8 points. A score of 2 or less is classified as no cognitive impairment (CN: cognitively normal), a score of 3–7 points is classified as mild cognitive impairment (MCI), and a score of 8 points or more is classified as dementia. This is a cognitive impairment evaluation tool that is not affected by education, age, or gender. At the time of the tool’s development, the sensitivity was 79% and the specificity was 80% [11].

### 2.3. Data Collection and Research Ethics

The survey on corporate and private taxi drivers working in Seoul was conducted with the Korea Transportation Safety Authority, Korea National Joint Conference of Taxi Association, Korea National Federation of Private Taxi Transportation Association, Korea National Federation of Taxi Workers’ Unions Federation, and Korea National Federation of Democratic Taxi Workers’ Unions.

The survey was approved by the Institutional Review Board/Ethnics committee of Konkuk University (IRB 7001355-202011-E-127). The survey subjects who agreed to the purpose of the study participated, and the survey period was from 22 August to 11 September 2018. The survey was conducted with a self-report method, with a total of 965 participants. Excluding five female respondents, a total of 960 people (501 corporate taxi drivers, 459 private taxi drivers) were analyzed.

### 2.4. Data Analysis

The collected data were analyzed using the statistical program R (R Foundation for Statistical Computing, Vienna, Austria). Descriptive statistics on physical, occupational, and sociocognitive characteristics of the study participants were presented as frequencies and percentages or means and standard deviations. The chi-square test and *t*-test were used to compare physical, occupational, and sociocognitive characteristics between the two groups of private and corporate taxi drivers. A significance level of 0.05 was set as the criterion for significance.

## 3. Results

### 3.1. Comparison of Physical Characteristics between Corporate and Private Taxi Drivers

Table 1 shows a comparison of the physical characteristics between corporate and private taxi drivers. The average age of private taxi drivers was 63.36 ± 7.67 years, which was higher than that of corporate taxi drivers, at 60.89 ± 7.76 years (*t* = −4.90, *p <* 0.001). The average BMI (kg/m^2^) was similar for corporate and private taxi drivers, at 24.64 ± 3.05 and 24.98 ± 2.62, respectively (*t* = −1.72, *p* = 0.086).

In the case of the percentage of those experiencing physical health discomfort in the last 2 weeks and the physical health discomfort period for the last 2 weeks, private taxi drivers revealed results of 5.4% and 0.24 ± 1.35 days, respectively, and corporate taxi drivers revealed results of 23% and 0.99 ± 2.36 days, respectively (χ^2^ = 54.40, *p <* 0.001; *t* = 5.89, *p* < 0.001). In other words, corporate taxi drivers had higher rates of physical health discomfort than private taxi drivers, and the period of physical health discomfort was longer than that for private taxi drivers.

The percentage of private taxi drivers who responded that their general physical health status was “bad” was only 3.7%, while the response rate of corporate taxi drivers was 22.3%, almost seven times higher than that of private taxi drivers (χ^2^ = 81.30, *p* < 0.001). The number of chronic diseases in corporate taxi drivers was 1.43 ± 1.87, whereas there were 0.76 ± 1.11 chronic diseases in private taxi drivers (*t* = 6.55, *p <* 0.001). The highest percentage of chronic diseases was occupied by hypertension (HTN), with 38.5% of corporate taxi drivers and 33.6% of private taxi drivers (χ^2^ = 2.29, *p* = 0.130). In addition, the rate of chronic obstructive pulmonary disease (COPD) and asthma was 1.1% for private taxi drivers, whereas it was 8.4% for corporate taxi drivers (χ^2^ = 25.14, *p* < 0.001).

### 3.2. Comparison of Occupational Characteristics between Corporate and Private Taxi Drivers

Table 2 shows a comparison of occupational characteristics between corporate and private taxi drivers. The taxi driving work experience was 13.86 ± 8.78 years for corporate taxi drivers and 24.20 ± 12.17 years for private taxi drivers (*t* = −15.15, *p <* 0.001). The number of working days per week was 5.89 ± 0.80 days for corporate taxi drivers and 4.60 ± 1.02 days for private taxi drivers (*t* = 20.94, *p <* 0.001). The average daily driving time and average daily mileage were 11.88 ± 2.80 days and 230.25 ± 68.51 km for private taxi drivers and 10.07 ± 1.52 days and 261.65 ± 85.24 km for corporate taxi drivers, respectively (*t* = −12.32, *p <* 0.001; *t* = 6.24, *p <* 0.001). The average number of night shifts per month was 10.14 ± 8.04 days for private taxi drivers and 12.36 ± 7.52 days for corporate taxi drivers (*t* = 4.23, *p <* 0.001).

The incidence rate and number of traffic accidents in the last 3 years were 28.5% and 1.70 ± 1.58 cases for private taxi drivers, respectively, and 51.0% and 2.00 ± 1.45 cases for corporate taxi drivers, respectively (χ^2^ = 45.27, *p* < 0.001; *t* = 1.68, *p* = 0.093). The incidence rate and number of near misses in the last 3 years were 27.7% and 2.72 ± 2.61 cases for private taxi drivers, respectively, and 51.5% and 4.23 ± 3.82 cases for corporate taxi drivers, respectively (χ^2^ = 45.71, *p* < 0.001; *t* = 3.47, *p* < 0.001). The percentage of those responding that traffic accidents (or near misses) and chronic diseases were “related” was 3.7% of private taxi drivers. On the other hand, 27.2% of corporate taxi drivers answered that traffic accidents (or near misses) and chronic diseases were “related”, which was seven times higher than that of private taxi drivers (χ^2^ = 74.95, *p* < 0.001).

### 3.3. Comparison of Sociocognitive Characteristics between Corporate and Private Taxi Drivers

Table 3 shows a comparison of sociocognitive characteristics between corporate and private taxi drivers. In terms of marital status, the percentage of those married was as high as 92.5% for private taxi drivers, but the rate of being single, divorced, or separated was as high as 29% for corporate taxi drivers (χ^2^ = 68.13, *p* < 0.001). The proportion of those with five or more dependent family members was 5.1% for private taxi drivers and 8.7% for corporate taxi drivers (χ^2^ = 7.48, *p* = 0.024). In terms of household monthly income, the ratio of household income of KRW 1–9 million a month was the highest for both corporate and private taxi drivers (χ^2^ = 14.30, *p* = 0.003). In terms of income satisfaction, the proportion of private taxi drivers who answered “there is not enough income” was 50%, whereas this result was over 70% for corporate taxi drivers (χ^2^ = 46.93, *p* < 0.001). The average score for cognitive function was 1.43 ± 0.71 points for corporate taxi drivers and 1.09 ± 0.32 points for private taxi drivers (*t* = 9.24, *p <* 0.001).

## 4. Discussion

This study attempted to present objective evidence for establishing health management policies for taxi drivers by comparing physical, occupational, and sociocognitive characteristics between corporate and private taxi drivers.

First of all, according to the physical characteristics of this study, the average age and proportion of taxi drivers over 70 years old were lower for corporate taxi drivers than private taxi drivers. Nevertheless, the percentage of corporate taxi drivers who experienced physical health discomfort in the last 2 weeks was higher than that of private taxi drivers, their general physical health status was worse than that of private taxi drivers, and the number of chronic diseases was also higher than that of private taxi drivers. It is thought that this is related to the occupational characteristics of corporate taxi drivers. In fact, as shown in this study, corporate taxi drivers had more working days per week and greater average daily mileage than private taxi drivers, as well as more frequent night shifts per month. This means that, although private taxi drivers were older than corporate taxi drivers, private taxi drivers can adjust their driving time according to their physical health status. On the other hand, corporate taxi drivers have to work shifts and pay the base payments to operate their taxis; thus, it is thought that there may be limitations in effectively managing the physical health problems of corporate taxi drivers. Corporate taxi drivers who are members of employee health insurance are considered to have a higher rate of diagnosis of chronic diseases through medical institutions because they are less burdened with medical expenses than private taxi drivers who are members of self-employed health insurance. In particular, it is thought that the rate of chronic diseases is relatively high in corporate taxi drivers because health checkups are essential to work as a corporate taxi driver. However, it is necessary to further investigate the onset and complications of chronic diseases in taxi drivers.

In fact, this study found that about 40% of corporate taxi drivers were overweight or obese with a BMI (kg/m^2^) of 25 or higher, and chronic diseases such as diabetes mellitus (DM), arthritis, and disc disease were higher in corporate taxi drivers than private taxi drivers. This is similar to the results of a previous study, where the rate of overweight and obese taxi drivers with a BMI (kg/m^2^) of 25 or higher reached 60–80% [12,13]. Furthermore, only 13% of taxi drivers had normal BMI and abdominal circumference [14]. In addition, the rates of DM and hyperlipidemia in taxi drivers were 19% and 34.6%, respectively [13], and the rate of those complaining of lower back pain reached 54% [15]. Increased age and BMI, as well as increased comorbid diseases such as hypertension (HTN), DM, and hyperlipidemia, have been reported to increase the risk of cardiovascular diseases (CVDs) such as myocardial infarction (MI) and angina [14,16]. In particular, MI or angina are thought to further increase the probability of unpredictable accidents due to the sudden onset of symptoms. However, it is thought that the subjective health status of taxi drivers according to the evaluation results of corporate taxi drivers’ physical health discomfort and general physical health status in this study can be an objective indicator to reduce the probability of unpredictable accidents as much as possible. Therefore, in preparing policies for taxi drivers, it is necessary to investigate not only objective information on taxi drivers’ various physical characteristics, but also subjective health status. In the domestic taxi industry, competition is intensifying due to the global economic crisis and the emergence of various platform transportation businesses [17,18,19,20]. Therefore, it is necessary to consider ways to encourage and implement lifestyle changes according to the situation of the domestic taxi industry.

In the case of occupational characteristics, as shown in this study, the number of working days per week and average daily mileage of corporate taxi drivers were greater, and the average number of night shifts per month was also higher. In fact, it was reported that corporate taxi drivers drive at a relatively faster speeds than private taxi drivers [8]. This is thought to be due to the obligation to pay the base payments to operate their taxis, as well as increased speed leading to higher income per unit time. This can be considered as increasing the risk of accidents for corporate taxis. In previous studies, it was reported that the daily driving time is a factor that affects the safety awareness of elderly commercial vehicle drivers [21]. Furthermore, while high-risk taxi drivers had longer taxi driving times, they had shorter breaks [22]. The traffic accidents caused by taxis lead to injuries and deaths among taxi drivers and passengers due to personal damage, and they are accompanied by enormous material damages [23]. In addition, even after traffic accidents, various psychological and emotional damages experienced by the person involved in the accident and their families are seen [24,25]. In particular, the damage from traffic accidents is not resolved in the short term and tends to be prolonged [24,25]. Therefore, it is thought that it is necessary to prepare for realistic policies such as a taxi total–pay–receipt system for the taxi industry to prevent repetitive traffic accidents by corporate taxi drivers. The taxi total–pay–receipt system, which was implemented in 2012, implements the abolition of the base payment by corporate taxi drivers and requires them to receive a salary [26]. Since 2020, it has been mandatory to implement the taxi total–pay–receipt system. Instead of using the base payment, the term “deposit” has been used, and, when this does not meet the standard, various illegal acts have been detected, such as deducting the monthly salary [27].

In the case of sociocognitive characteristics, this study found that corporate taxi drivers had a higher number of dependent family members, lower monthly household income, and lower income satisfaction, despite having no spouse.

In fact, corporate taxi drivers had increased working days per week and daily mileage, as well as a high percentage of late-night driving, but this was found to be less than 50% of the target actual business time rate of the Ministry of Land, Infrastructure, and Transport [8]. The actual business time rate is a value obtained by dividing taxi business hours (the hours during which a taxi was driven with passengers) by taxi operating (or driving) time (the hours during which a taxi was driven with passengers + the hours during which the taxi was driven without any passenger) and multiplying by 100. In particular, it is reported that the actual business time rate is only about 60%, despite the increase in the supply of taxis during times of high demand for taxis [8].

The financial burden of taxi drivers leads to excessive driving tasks, which in turn increases the fatigue of the taxi driver. In fact, more than 60% of taxi drivers report fatigue [28]. In addition, it is reported that the economic burden of taxi drivers affects their safe driving behavior [29,30], and at least 21% of drivers in fatal accidents reported experiencing sleepiness while driving [31]. In the case of corporate taxis, they are workers under the Labor Standards Act, and there is no problem when they are involved in occupational accidents because they are workplaces subject to occupational accidents. However, in the case of private taxis, occupational accidents can be applied only if they have voluntarily joined the Korea Workers’ Compensation and Welfare Service with approval [32]. In the case of taxi drivers, when a traffic accident occurs, most of them are covered by auto insurance rather than occupational accidents. This is because, if taxi drivers treat a traffic accident as an occupational accident, the occupational accident insurance rate may increase, and the taxi company may be subject to supervision by the Labor Office as a workplace with frequent occupational accidents [32].

In fact, after the financial crisis and global economic crisis, the number of vulnerable healthcare groups in Korea increased, and healthcare blind spots further expanded in providing healthcare services. In particular, the low-income class has limited access to healthcare services due to economic factors, and the health gap between economic classes widened further [33].

On another note, hydrogen and electric vehicles, which are showing an increasing trend all over the world, generate less noise and are environmentally friendly [34]. By 2035, the expansion of government support for converting hydrogen and electric vehicles is planned, focusing on public transportation, among business vehicles [35]. However, in recent years, due to such low noise, safety issues for drivers and pedestrians have emerged [34]. Therefore, it is considered that laws and policies should be prepared to prevent the occurrence of various accidents caused by taxis, one of the means of public transportation.

As shown in this study, the rates of mild cognitive impairment and dementia were significantly higher for corporate taxi drivers. Although it is reported that the incidence of cognitive impairment increases with age [36,37], various factors are thought to have an influence on cognitive impairment. In fact, physical and socioeconomic factors are reported to affect traffic accidents and driving behavior, along with cognitive function [29,30]. Therefore, it is thought that the physical health status and socioeconomic burden of taxi drivers indirectly influenced the degree of cognitive impairment. Since this study did not investigate the psychological health status of taxi drivers, a comprehensive survey of the physical and psychological health status of taxi drivers is considered necessary in the future. Moreover, it is reported that higher social support from the spouse and family leads to a higher likelihood of returning to work after a traffic accident [38]. In particular, as shown in this study, since the proportion of marriages with spouses was lower for corporate taxi drivers than for private taxi drivers, the opinions of spouses and family members need to be reflected in the healthcare policy of taxi drivers.

Since this study had a problem of selection bias due to non-probability sampling, as well as excluding female taxi drivers in the selection of study subjects, there is a limit to generalizing the findings to the characteristics of all corporate and private taxi drivers. However, the number of subjects for this study was not small, and, while it was limited to males, there was no significant difference in the frequency of the number of subjects by age group. Therefore, it can be said that it was a very meaningful approach, in that it explored the problems faced by corporate and private taxi drivers through a comparison of physical, occupational, and sociocognitive characteristics.

## 5. Conclusions

In this study, we compared various physical, occupational, and sociocognitive characteristics of corporate and private taxi drivers in Seoul. According to the results of this study, the below suggestions are made.

First, it is thought that taxi drivers 65 years of age or older should receive additional health checkups, as well as computer simulation tests conducted to maintain the qualifications of taxi drivers. In particular, it is believed that health checkups for taxi drivers over 65 years of age should be legally mandated to expand public healthcare. Furthermore, for taxi drivers under the age of 65, it is thought that it is necessary to consider methods such as subjective physical and psychological health status evaluations and cognitive dysfunction assessments to replace health checkups.

Second, it is thought that there is a need to prepare an institutional and legal arrangement to reduce the economic burden of taxi drivers. In 2015, the Seoul Metropolitan Government began a policy of obligating a digital tachograph (DTG) to be installed in all taxis (100%) in Seoul. However, since DTGs have not yet been installed in other regions outside Seoul, it is necessary to try to solve the problem with the expansion of DTG use in other regions, along with the analysis of large-scale DTG data.

Ultimately, this comparison of the characteristics of corporate and private taxis is expected to serve as evidence for developing tailored policies and programs to improve the health of corporate and private taxi drivers, ensuring the safety of the public.

## Figures and Tables

**Table 1:** Physical characteristics of corporate and private taxi drivers.

Item	Corporate Taxi	Private Taxi	*t* or χ^2^	*p*
	M ± SD or *N* (%)	M ± SD or *N* (%)		
No. of subjects	501 (52.2)	459 (47.8)		
Age	60.89 ± 7.76	63.36 ± 7.67	−4.90	<0.001
≤40s	38 (7.8)	22 (4.9)	16.72	<0.001
50s	142 (29.0)	102 (22.9)		
60s	254 (51.8)	234 (52.5)		
≥70s	56 (11.4)	88 (19.7)		
BMI (kg/m^2^)	24.64 ± 3.05	24.98 ± 2.62	−1.72	0.086
<25	255 (59.3)	218 (51.4)	6.45	0.040
25–30	158 (36.8)	192 (45.3)		
≥30	17 (3.9)	14 (3.3)		
Physical health discomfort in the last 2 weeks				
Yes	106 (22.6)	24 (5.4)	54.40	<0.001
The duration of health discomfort in the last 2 weeks (days)	0.99 ± 2.36	0.24 ± 1.35	5.89	<0.001
General physical health status				
Good and very good	177 (35.6)	251 (54.9)	81.30	<0.001
Usual	209 (42.1)	189 (41.4)		
Bad and very bad	111 (22.3)	17 (3.7)		
Taxi driving effect of having chronic diseases				
Not affected	217 (56.5)	280 (84.9)	95.60	<0.001
Usually	56 (14.5)	42 (12.7)		
Influential	11 (29.0)	8 (2.4)		
Number of chronic diseases	1.43 ± 1.87	0.76 ± 1.11	6.55	<0.001
Types of chronic diseases				
HTN				
Yes	188 (38.5)	151 (33.6)	2.29	0.130
DM				
Yes	123 (25.2)	73 (16.2)	10.89	0.001
COPD and asthma				
Yes	41 (8.4)	5 (1.1)	25.14	<0.001
Sleep disorder				
Yes	46 (9.4)	7 (1.6)	25.75	<0.001
Anxiety, depression, and panic disorder				
Yes	24 (4.9)	5 (1.1)	10.09	0.001
Hearing loss				
Yes	35 (7.2)	13 (2.9)	7.99	0.005
Arthritis				
Yes	77 (15.8)	19 (4.2)	32.79	<0.001
Disc disease				
Yes	70 (14.3)	28 (6.2)	15.65	<0.001

M = mean; SD = standard deviation; BMI = body mass index; HTN = hypertension; DM = diabetes mellitus; COPD = chronic obstructive pulmonary disease.

**Table 2:** Occupational characteristics of corporate and private taxi drivers.

Item	Corporate Taxi	Private Taxi	*t* or χ^2^	*p*
	M ± SD or *N* (%)	M ± SD or *N* (%)		
Taxi driving work experience (years)	13.86 ± 8.78	24.20 ± 12.17	−15.15	<0.001
Driving frequency (days/week)	5.89 ± 0.80	4.60 ± 1.02	20.94	<0.001
Daily driving time(h/day)	10.07 ± 1.52	11.88 ± 2.80	−12.32	<0.001
Daily mileage (km)	261.65 ± 85.24	230.25 ± 68.51	6.24	<0.001
Number of night shifts (per month)	12.36 ± 7.52	10.14 ± 8.04	4.23	<0.001
Traffic accidents in the last 3 years				
Yes	229 (51.0)	122 (28.5)	45.27	<0.001
Near misses in the last 3 years				
Yes	211 (51.5)	106 (27.7)	45.71	<0.001
The number of traffic accidents in the last 3 years	2.00 ± 1.45	1.70 ± 1.58	1.68	0.093
1	115 (51.1)	70 (64.8)	12.42	0.006
2	44 (19.6)	24 (22.2)		
3	47 (20.9)	7 (6.5)		
≥4	19 (8.4)	7 (6.5)		
The number of near misses in the last 3 years	4.23 ± 3.82	2.72 ± 2.61	3.47	<0.001
1–2	96 (47.8)	62 (66.0)	61.29	<0.001
3–4	32 (15.9)	13 (13.8)		
5–6	28 (13.9)	12 (12.8)		
≥7	45 (22.4)	7 (7.4)		
Relationship between having chronic diseases and traffic accidents or near misses				
Not relevant	269 (64.8)	292 (89.3)	74.95	<0.001
Usually	33 (8.0)	23 (7.0)		
Relevant	113 (27.2)	12 (3.7)		

**Table 3:** Sociocognitive characteristics of corporate and private taxi drivers. KRW, South Korean won.

Item	Corporate Taxi	Private Taxi	*t* or χ^2^	*p*
	M ± SD or *N* (%)	M ± SD or *N* (%)		
Marital status				
Married (having a spouse)	344 (71.0)	407 (92.5)	68.13	<0.001
Other (single, separated, divorced, etc.)	140 (29.0)	33 (7.5)		
Number of dependent family member (adulthood and underage)	2.84 ± 1.20	2.67 ± 1.04	2.26	0.024
1–2	204 (49.1)	236 (57.3)	7.48	0.024
3–4	175 (42.2)	155 (37.6)		
≥5	36 (8.7)	21 (5.1)		
Household income (per month)				
<100 (KRW 10,000)	93 (21.7)	62 (15.0)	14.30	0.003
100–199 (KRW 10,000)	218 (51.0)	198 (47.9)		
200–299 (KRW 10,000)	83 (19.4)	94 (22.8)		
≥300 (KRW 10,000)	34 (7.9)	59 (14.3)		
Income Satisfaction				
Enough	11 (2.2)	22 (4.9)	46.93	<0.001
Average	126 (25.7)	201 (44.7)		
Not enough (insufficient)	354 (72.1)	227(50.4)		
Cognition	1.43 ± 0.71	1.09 ± 0.32	9.24	<0.001
CN	348 (70.2)	412 (91.8)	78.73	<0.001
MCI	83 (16.7)	33 (7.3)		
Dementia	65 (13.1)	4 (0.9)		

KDSQ (Korean Dementia Screening Questionnaire); CN = cognitively normal (KDSQ ≤ 2); MCI = mild cognitive impairment (KDSQ 3–7); dementia (KDSQ ≥ 8). One million KRW = 895 United states dollars (USD) = 766 euros (EUR), according to exchange rate as of 2018.

## Data Availability

Data were obtained from a secondary provider and are not publicly available.
